# Cis-regulation of *FAM49A* by a risk variant at 2p24 contributes to the genetic susceptibility of NSCL/P

**DOI:** 10.1016/j.gendis.2025.101875

**Published:** 2025-09-30

**Authors:** Junyan Lin, Xiaofeng Li, Ji Mao, Xinyu Zhang, Tingting Cheng, Yue Gao, Shu Lou, Dandan Li, Yongchu Pan

**Affiliations:** a Department of Orthodontics, The Affiliated Stomatological Hospital of Nanjing Medical University, Nanjing, Jiangsu 210029, China; b State Key Laboratory Cultivation Base of Research, Prevention and Treatment for Oral Diseases (Nanjing Medical University), Nanjing, Jiangsu 210029, China; c Jiangsu Province Engineering Research Center of Stomatological Translational Medicine (Nanjing Medical University), Nanjing, Jiangsu 210029, China

Non-syndromic cleft lip with or without cleft palate (NSCL/P) represents the most common craniofacial malformation worldwide, with an incidence of approximately 1:700 live births. Summary-data-based Mendelian randomization (SMR) integrative analysis of genome-wide association study (GWAS) and expression quantitative trait loci (eQTL) summary data identified *FAM49A* as an NSCL/P risk gene. The variant rs4240230 (PP4 = 0.99) exhibited causal effects on NSCL/P risk and *FAM49A* expression in Bayesian co-localization. Chromatin conformation capture (3C) revealed that the rs4240230 locus exhibits frequent physical interactions with the *FAM49A* gene, and the enhancer activity was validated by chromatin immunoprecipitation (ChIP) and CRISPR activation (CRISPRa). The G allele recruited less HLTF and reduced enhancer activity compared with the A allele in dual-luciferase reporter assays, ChIP and electrophoretic mobility shift assays (EMSAs). *FAM49A* deficiency inhibited proliferation and increased apoptosis and migration in mouse cranial neural crest cells (O9-1) while disrupting collagen fibril organization, extracellular matrix organization and bone development pathways. Deficient *fam49a* expression in zebrafish led to shortened body length, spinal curvature, ethmoid plate defects, and elevated mortality/deformity. In conclusion, the variant G allele of rs4240230 reduced HLTF binding affinity to *FAM49A*, suppressed *FAM49A* expression, and contributed to NSCL/P susceptibility.

NSCL/P is a complex developmental disorder with heterogeneous genetic and environmental contributions. Despite advances in GWASs, identifying causal variants and their functional mechanisms remains challenging due to extensive linkage disequilibrium. Here, by applying SMR analysis to our previous GWAS^1^ (1069 cases and 1724 controls) and eQTLgen datasets, we identified *FAM49A* as a novel NSCL/P risk gene (Fig. 1A). Co-localization analysis identified significant genetic overlap between NSCL/P GWAS loci and blood eQTL signals, with rs4240230 emerging as a shared causal variant (PP4 = 0.99). This SNP showed dual associations: (1) elevated NSCL/P susceptibility (GWAS effect in additive model: OR = 1.50, 95% CI = 1.36–1.64, *p* = 6.60E-09), and (2) transcriptional regulation of *FAM49A* in whole blood (eQTL effect: *p* = 3.64E-39) (Fig. 1B and Table S1).

**Figure 1:**
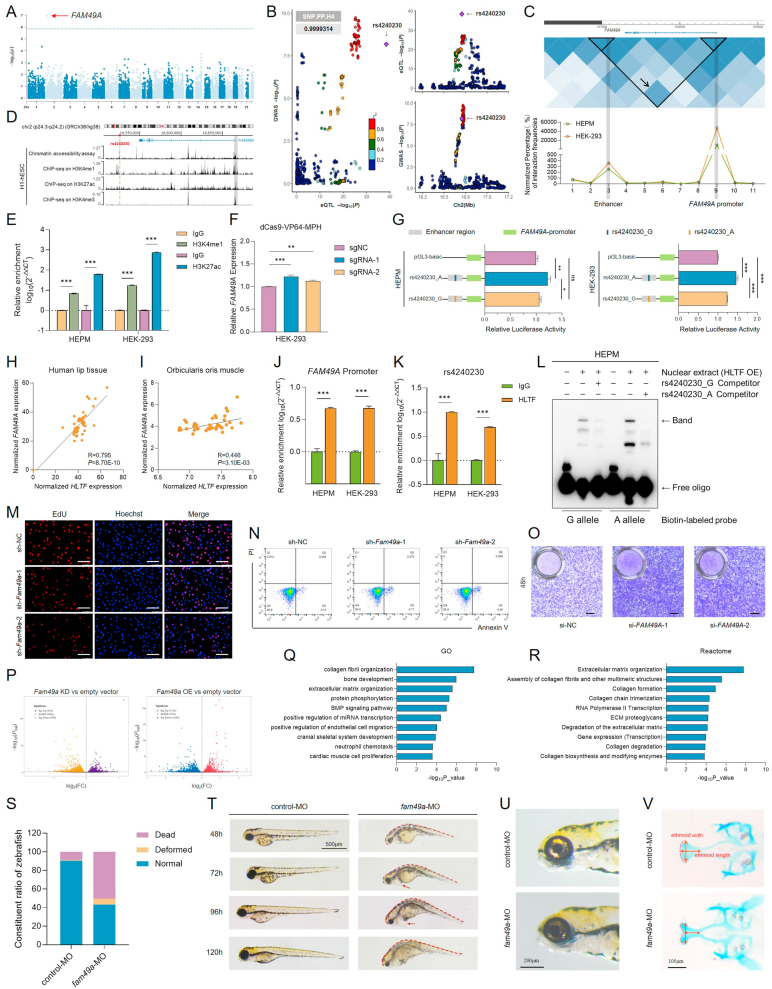
Cis-regulation of FAM49A by a risk variant at 2p24 contributed to the genetic susceptibility of NSCL/P. **(A)** Manhattan plot of SMR showed the causal association between genetic variants and NSCL/P. Dashed line: *P*_FDR_ = 0.05/14610. **(B)** Scatterplot of GWAS and eQTL associations for rs4240230. The left plot showed the association between the rs4240230 GWAS and eQTL. The upper right plot showed the eQTL results of rs4240230 and its linked sites in whole blood. The lower right plot showed the association results of rs4240230 and its linked sites in the NSCL/P GWAS. **(C)** Hi-C data from the GEO database (GSE18199) were visualized using the WashU browser. Different colors indicated different interaction frequencies. The dark blue region indicated by the black arrow represents high-frequency physical interactions between the genomic region containing rs4240230 and the promoter of *FAM49A*. 3C experiments validated chromatin interactions spanning rs4240230 to the *FAM49A* promoter following *BstNI* restriction enzyme digestion. **(D)** Chromatin accessibility assay and ChIP-seq signals for human embryonic stem cells from the GEO database (GSE18927 and GSE16356) showed that the rs4240230 locus resides within the DHS and exhibits enrichment of H3K4me1 and H3K27ac histone modifications. **(E)** ChIP–qPCR illustrated the enrichment of H3K4me1 and H3K27ac on rs4240230 in HEPM and HEK-293 cells. **(F)** Inducible overexpression of *FAM49A* by RT–qPCR in dCas9-VP64-MPH cells. sgRNA-1 and sgRNA-2 were designed to target the rs4240230-containing genomic region, and no-targeting sgNC was used as a negative control. **(G)** Luciferase assay was performed in HEPM and HEK-293 cells to detect the transcriptional activity of *FAM49A*. **(H, I)** Correlation analysis between *HLTF* and *FAM49A* in 40 lip tissues (H) and the orbicularis oris muscle (I, GSE85748). **(J, K)** ChIP–qPCR illustrated the enrichment of HLTF on the *FAM49A* promoter (J) and the rs4240230 region (K) in HEPM and HEK-293 cells. The binding of HLTF is shown as relative enrichment over that of IgG. **(L)** The binding affinities between oligos containing the G allele or A allele of rs4240230 and *FAM49A* were analyzed by EMSA. HLTF OE: nuclear proteins extracted from HLTF-overexpressing HEPM cells. **(M)** Representative fluorescence microscopy images showing EdU incorporation (proliferation) in O9-1 cells. Scale bar: 100 μm. **(N)** Representative flow cytometry dot plots showing apoptosis in O9-1 cells. **(O)** Representative images of migrated O9-1 cells on the lower membrane surface in transwell assays. Scale bar: 100 μm. **(P)** Volcano plot of differentially expressed genes between *Fam49a* knockdown or overexpression in O9-1 cells. (KD, knockdown; OE, overexpression.) **(Q, R)** GO analysis (Q) and reactome analysis (R) of the DEGs. **(S)** Statistical analysis of the number of dead, deformed or normal embryos. **(T)** Representative lateral views of 48–120 hpf zebrafish larvae injected with control-MO or *fam49a*-MO. Spinal curvature phenotypes are demarcated by red dashed lines, while pericardial edema is indicated with red arrows. Scale bar: 500 μm. **(U)** Iridophore pigmentation in 120 hpf embryos. Scale bar: 200 μm. **(V)** Alcian blue-stained ethmoid cartilage in 120 hpf embryos. Scale bar: 100 μm. The results were presented as the mean ± SD of three independent experiments. ns, not significant; ∗*p* < 0.05, ∗∗*p* < 0.01, or ∗∗∗*p* < 0.001 indicates a significant difference between the groups.

To distinguish whether the overlap between eQTL and GWAS signals is a shared causal variant, functional validation of eQTL regulation for *FAM49A* with NSCL/P was performed. Frequent physical interactions between the rs4240230-containing region and the *FAM49A* promoter, identified via Hi-C profiling of human lymphoblastoid cell line (GSE18199), were verified by 3C assays of human embryonic palatal mesenchymal (HEPM) cells and human embryonic kidney 293 (HEK-293) cells (Fig. 1C). Rs4240230 is located at approximately 140 kb downstream of the transcription start site (TSS) of *FAM49A.* The locus harboring rs4240230 exhibited concurrent DNase I hypersensitive sites (DHSs) and H3K4me1 and H3K27ac occupancies, as demonstrated by a chromatin accessibility assay and ChIP-seq profiling of human embryonic stem cells (H1-hESCs) in a public dataset (GSE18927 and GSE16356, Fig. 1D), consistent with canonical enhancer features. ChIP assays in both HEPM and HEK-293 cells revealed co-enrichment of H3K4me1 and H3K27ac at the genomic region encompassing rs4240230, confirming its localization within the active enhancer element (Fig. 1E; Fig. S1A, S1B). Targeted activation of the rs4240230-containing region via the dCas9-VP64-MPH system in HEK-293 cells increased *FAM49A* transcript levels, demonstrating enhancer activity at this locus for *FAM49A* (Fig. 1F). Compared with the rs4240230-A allele, the G allele had lower enhancer activity for *FAM49A* in HEPM and HEK-293 cells in dual luciferase reporter assays (Fig. 1G).

Transcription factors (TFs) are essential for the function of enhancers as cis-regulatory elements. The differential TF binding affinity of the rs4240230 A/G allele might be responsible for differences in enhancer activity.^2^ The top 10 TFs exhibiting the most significant binding differences between the A and G alleles were predicted via the PERFECTOS-APE online platform (Fig. S1C and Table S2). Notably, *HLTF* demonstrated consistent positive correlations with *FAM49A* in both the lip tissue^3^ (*r* = 0.795, *p* = 8.70E-10) and orbicularis oris muscle tissue (*r* = 0.446, *p* = 3.10E-03, GSE85748) datasets (Fig. 1H, I). ChIP-seq profiling in K562 cells (GSE91466) revealed HLTF binding at both the rs4240230 and *FAM49A* promoter regions (Fig. S1D), which was further validated through ChIP assays in HEPM and HEK-293 cells (Fig. 1J, K; Fig. S1E). Nuclear extracts from HEPM cells overexpressing HLTF showed lower binding efficiency with the rs4240230-G allele than with the A allele in EMSA (Fig. 1L). Besides, *HLTF* knockdown led to decreased expression of *FAM49A* (Fig. S1F). These findings demonstrated that HLTF exhibited preferential binding to the rs4240230-A allele, which increased the enhancer activity for *FAM49A* transcription.

The enhancer activity of rs4240230 suggested three-dimensional proximity between this locus and the *FAM49A* promoter region. CCCTC-binding factor (CTCF) is a protein enriched at loop anchors and is critical for the formation and maintenance of chromatin loops.^4^ By analyzing data from the Gene Expression Omnibus (GEO) database (GSE31477, GSE29611, and GSE33213), we identified abundant CTCF binding sites near the *FAM49A* promoter and the rs4240230 region (Fig. S2A). Furthermore, these regions reside within a topologically associating domain (TAD) and exhibit multiple potential chromatin looping interactions in human pluripotent cells (GSE69647, Fig. S2A). To validate the role of CTCF in chromatin loop formation, we knocked down *CTCF* in HEPM and HEK-293 cells and observed decreased *FAM49A* expression (Fig. S2B, S2C). These results indicated that CTCF mediated chromatin loop formation to establish spatial proximity between regulatory elements, thereby activating *FAM49A* gene expression.

*FAM49A*, also known as *CYRIA*, was associated with NSCL/P in a multi-ethnic GWAS of 6480 participants with European, Asian, African and Central and South American ancestry in 2016.^5^ Subsequent studies have consistently replicated associations between NSCL/P susceptibility and variants within the *FAM49A* locus. We investigated the functional role of *FAM49A in vitro*. EdU incorporation and flow cytometry-based apoptosis analysis revealed that *FAM49A* knockdown in HEPM cells (Fig. S3A) significantly inhibited proliferation (Fig. S3B) and increased apoptosis (Fig. S3C). Furthermore, transwell migration assays demonstrated accelerated migration capacity in *FAM49A*-knockdown HEPM cells (Fig. S3D).

scRNA-seq clustering of 18 h post-fertilization (hpf) zebrafish embryogenesis from the ZESTA database revealed *fam49a* expression in neural crest cell clusters, a key progenitor population essential for craniofacial development (Fig. S4A, S4B). Consistent with the phenotypic alterations in HEPM cells, reduced *Fam49a* expression in O9-1 cells (Fig. S4C) resulted in suppressed proliferation (Fig. 1M), increased apoptosis (Fig. 1N), and accelerated migration (Fig. 1O).

To further explore the function of *FAM49A*, differential gene expression by knockdown or overexpression of *Fam49a* in O9-1 cells was selected (Fig. S4D, S4E). *Fam49a* knockdown led to the differential expression of 1793 genes (834 up-regulated and 959 down-regulated, *p* < 0.05), while *Fam49a* overexpression led to the differential expression of 3382 genes (1754 up-regulated and 1628 down-regulated, *p* < 0.05) (Fig. 1P). The intersection between genes up-regulated upon *Fam49a* overexpression and those down-regulated upon *Fam49a* knockdown in O9-1 cells comprised 21 genes, and the overlap between genes down-regulated upon *Fam49a* overexpression and those up-regulated upon *Fam49a* knockdown comprised 44 genes (Fig. S4F). Gene Ontology (GO) analysis and reactome analysis showed that these two cohorts of genes were mainly related to collagen fibril organization, extracellular matrix organization, and bone development processes (Fig.1Q, R).

Zebrafish is a commonly used model for studying craniofacial development. *fam49a*-knockdown zebrafish (Fig. S4G) demonstrated high death rate and malformation rate (Fig. 1S), with deformities including spinal curvature and heart-associated edema (Fig. 1T). Meanwhile, these zebrafish exhibited decreased iridophores (Fig. 1U), a terminal cell type derived from neural crest cells, indicating impaired neural crest development. Additionally, *FAM49A*-deficient zebrafish displayed underdeveloped ethmoid plate dimensions (width and length) in craniofacial regions (Fig. 1V; Fig. S4H, S4I), phenocopying human maxillary developmental anomalies such as NSCL/P.

Taken together, our study delineated a regulatory mechanism whereby rs4240230 modulated *FAM49A* expression through allele-specific enhancer activity, contributing to NSCL/P susceptibility (Fig. S5).

## CRediT authorship contribution statement

**Junyan Lin:** Writing – original draft, Conceptualization. **Xiaofeng Li:** Formal analysis, Conceptualization. **Ji Mao:** Validation. **Xinyu Zhang:** Data curation. **Tingting Cheng:** Visualization. **Yue Gao:** Methodology. **Shu Lou:** Writing – review & editing. **Dandan Li:** Writing – review & editing. **Yongchu Pan:** Writing – review & editing, Conceptualization.

## Ethics declaration

Ethical compliance was approved by the Ethics Committee of Nanjing Medical University (NJMUERC [2008] No. 20), with written informed consent obtained from all participants.^4^

## Data availability

The authors declare that all other data are contained within the files or available on request.

## Funding

This work was supported by the National Natural Science Foundation of China (Nos. 82270946, 82571049), the Jiangsu Province Capability Improvement Project through Science, Technology and Education-Jiangsu Provincial Research Hospital Cultivation Unit (China) (No. YJXYYJSDW4), Jiangsu Provincial Medical Innovation Center (China) (No. CXZX202227), and the Dental Disease Cohort Project of Affiliated Stomatological Hospital, Nanjing Medical University, Jiangsu, China.

## References

[1] Lou S., Zhu G., Xing C. (2024). Transcriptome-wide association identifies KLC1 as a regulator of mitophagy in non-syndromic cleft lip with or without palate. Imeta.

[2] Hua J.T., Ahmed M., Guo H. (2018). Risk SNP-mediated promoter-enhancer switching drives prostate cancer through lncRNA PCAT19. Cell.

[3] Li X., Tian Y., Qiu L. (2022). Expression quantitative trait locus study of non-syndromic cleft lip with or without cleft palate GWAS variants in lip tissues. Cells.

[4] Karpinska M.A., Zhu Y., Fakhraei Ghazvini Z. (2025). *CTCF* depletion decouples enhancer-mediated gene activation from chromatin hub formation. Nat Struct Mol Biol.

[5] Leslie E.J., Carlson J.C., Shaffer J.R. (2016). A multi-ethnic genome-wide association study identifies novel loci for non-syndromic cleft lip with or without cleft palate on 2p24.2, 17q23 and 19q13. Hum Mol Genet.

