# Supplementary material for: Cis-regulation of FAM49A by a risk variant at 2p24 contributes to the genetic susceptibility of NSCL/P
Source: Genes Dis. 2025 Sep 30;13(4):101875. doi: 10.1016/j.gendis.2025.101875 (PMC13015221; doi:10.1016/j.gendis.2025.101875)
Supplement: Multimedia component 1 [file mmc1.docx]

**Cis-regulation of *FAM49A* by risk variant at 2p24 contributed to genetic susceptibility of NSCL/P**

**Supplementary Data**

**Material and Methods**

**Summary-data-based Mendelian randomization (SMR) analysis**

The summary statistics was extracted from our previous genome-wide association study (GWAS) of Non-syndromic cleft lip with or without cleft palate (NSCL/P) compromising 1,069 cases and 1,724 controls^[1]^. Expression quantitative trait loci (eQTL) summary data was from eQTLGen Consortium Phase 1 database containing eQTL information from human whole-blood or peripheral blood monouclear cells (PBMC) samples (n=31,684)^[2]^.

To investigate putative causal relationship between genes and NSCL/P risk, SMR analysis was conducted under a fixed-effects inverse-variance weighted model by the SMR software (v1.3.1). cis-eQTL genetic variants were employed as instrumental variables, assigning gene expression to exposure and NSCL/P to outcome. Bonferroni correction was applied to account for multiple testing (threshold=0.05/14610).

**Co-localization of GWAS signals and eQTL**

Bayesian co-localization was conducted to perform genetic colocalization of GWAS and eQTL data using the package “coloc” in R 4.3.3 software. The analysis included 5 posterior probabilities: PP0: causal variant for neither GWAS nor eQTL; PP1: only GWAS is significant. PP2: only eQTL is significant. PP3: GWAS and eQTL driven by different causal variations. PP4: GWAS and eQTL driven by the same causal variant. If PP4 ≥ 0.8, the NSCLP and eQTL were considered to be driven by the same SNP. The regional plots were drawn with LocusCompare. (http://locuscompare.com/)^[3]^.

**Cell culture**

The human embryonic palatal mesenchymal (HEPM) cells (CRL-1486, ATCC, Manassas, VA, USA) and human embryonic kidney 293 (HEK-293) cells (CRL-1573, ATCC, Manassas, VA, USA) were purchased from the American Type Culture Collection (ATCC) and respectively maintained in α-MEM (Gibco, USA) and DMEM (Gibco, USA) supplemented with 10% fetal bovine serum (FBS) (Gibco, USA) and 1% penicillin-streptomycin (PS) (C0222, Beyotime, China).

The mouse cranial neural crest cells (O9-1) (SCC049, Merck, Germany) were maintained in mouse ES cell basal medium (SCRR-2011; ATCC, USA) supplemented with 10% FBS, 1% PS, 0.03% leukemia inhibitory factor (ESG1106, Sigma, Darmstadt, Germany) and 0.18% 2-Mercaptoethanol (21985-023, Gibco, USA).

**Chromatin conformation capture (3C)**

HEPM and HEK-293 cells were crosslinked by 1% formaldehyde at room temperature. After 10 minutes, glycine was added to terminate the crosslinking reaction. The cell pellet was lysed with 0.3% SDS and 2% TritonX-100 and then digested with restriction endonuclease BstNI (ER0551, Thermo Scientific, USA) at 37°C overnight. The suspension was placed at 65°C for 1h with shaking. Next, the DNA fragments were ligated by T4 ligase (D7006, Beyotime, China) in buffer R (ER0551, Thermo Scientific, USA) at 25°C for 4h, and 200μg proteinase K was added to digest the proteins surrounding the target DNA. Finally, the ligation products were purified by DNA purification kits and subjected to RT-qPCR. Sequences of primers are in **Table S3**.

**Chromatin immunoprecipitation (ChIP) assay**

ChIP assays were conducted using the Magna ChIP^TM^ Kit (17-10085, 17-10086, Magna, Germany) followed manufacturer’s introductions. In brief, formaldehyde was added to the culture medium, and the cells were incubated at room temperature to crosslink the DNA and proteins. After 10 minutes, glycine was added to terminate the crosslinking reaction. Next, the cell membrane and nuclear membrane were lysed to obtain chromatin, which was fragmented into 200-1000 bp segments using sonication. The chromatin fragments were incubated overnight at 4°C with antibodies of H3K27ac (ab4729, Abcam, Cambridge, UK), H3K4me1 (ab176877, Abcam) and HLTF (14286-1-AP, Proteintech, Wuhan, China). DNA was purified using a DNA purification kit (D0033, Beyotime, China) on the following day. RT-qPCR and agarose gel electrophoresis were then performed (primers listed in **Table S3**).

**CRISPR activation (CRISPRa) of rs4240230**

We constructed a HEK-293 cell line with stable expression of dCas9-VP64 fusion protein and MS2-P65-HSF1(MPH) activation auxiliary protein (dCas9-VP64-MPH). The cells were maintained with blasticidin (10 μg/mL) and hygromycin (200 μg/mL). Two sgRNAs targeting rs4240230-containing genomic region were designed. The sgRNA associated with dCas9-VP64 can bind to the target DNA sequence, recruit the MPH complex and RNA polymerase (RNAP), then activate the target region. The sgRNA sequences are in **Table S3**.

**Dual Luciferase reporter assay**

The plasmids were co-transfected with pRL-SV40 and Lipofectamine 2000 reagent into HEPM and HEK-293 cells. After 24h, transfected cells were lysed using Dual Luciferase Reporter Assay Kit (DL101-01, Vazyme, Nanjing, China) and centrifuged at 10,000 ×g for 5 min at 4℃. 20 μL of supernatant was collected for quantifying the ratio of Firefly to Renilla luciferase. Sequences of plasmids are in **Table S3**.

**Cell transfection**

Gene-specific small interfering RNAs (siRNAs) targeting human *FAM49A*, *HLTF*, and *CTCF* were synthesized (GenePharma, Shanghai, China). Transient transfection of HEPM and HEK-293 cells was performed using Lipofectamine 2000 reagent (11668019, Invitrogen, Carlsbad, CA, USA).

O9-1 cells were prepared and stably infected at a multiplicity of infection (MOI) of 20 with sh-*Fam49a* vector lentiviruses or *Fam49a*-overexpressing lentiviruses (GenePharma) by polybrene. After 48h, cells were selected with puromycin (1299MG025, BioFroxx, Germany). The sequences of the siRNAs and shRNAs are in **Table S3**.

**Electrophoretic mobility shift assay (EMSA)**

Unlabeled competitor probes and biotin-labeled oligonucleotide probes corresponding to the A or G alleles were designed and synthesized (**Table S3**). Nuclear proteins of HEPM cells were co-incubated with the probes according to the instructions of the Chemiluminescent EMSA Kit (20158, Thermo Scientific, USA). Protein-DNA complexes were resolved on 6.5% non-denaturing polyacrylamide gels in 0.5 × TBE buffer electrophoresed at 100 V for 90 minutes, and transferred to a nylon membrane at 380 mA for 45 minutes. After crosslinked by UV light for 12 minutes, the membranes were blocked, conjugated, washed, and balanced in sequence. Binding reactions were detected using a Chemiluminescent Substrate.

**EdU incorporation assay**

After transfection, cells were pulsed to 50 μM 5-ethynyl-2’-deoxyuridine (EdU) (C10310-1, Ribobio, Guangzhou, China) at 37°C for 2h. Following fixation with 4% paraformaldehyde (PFA) for 30 minutes and permeabilization with 0.1% Triton X-100 for 10 minutes at room temperature, EdU-positive nuclei were fluorescently labeled via copper-catalyzed click chemistry.

**Cell apoptosis assay**

The cells were collected and washed with PBS. After adding 100 μL binding buffer and 2 μL Annexin V-FITC or Annexin V-PE, the cell suspension was incubated on ice for 15 minutes in the dark. The cell suspension was filtered through a 40-μm cell strainer into flow cytometry tubes, where 400 μL of PBS and 1 μL propidium iodide (PI) were added to each tube; the mixture was then incubated for 2 minutes at room temperature protected from light and immediately analyzed using a flow cytometer. FlowJoX software was used for analysis.

**Cell migration assays**

High-serum culture medium containing 20% FBS was added to a 24-well plate. Transfected cells were seeded onto transwell inserts (CLS3422, Corning, Germany) in a normal complete medium. The chamber was positioned within the 24-well plate, ensuring the high-serum medium completely immerses the insert membrane. After incubation for 24h to 48h, migrated cells were fixed with 4% PFA for 30 minutes and stained with crystal violet (C0121, Beyotime, China) for 30 minutes. The membrane bottom was photographed under a stereo microscope for migrating cell quantification.

**RNA-sequencing**

Surgical surplus lip tissues (n=40) were collected from NSCL/P patients. Ethical compliance was approved by the Ethics Committee of Nanjing Medical University (NJMUERC [2008] No. 20), with written informed consent obtained from all participants^[4]^.

Total RNA isolation was extracted by the TRIzol method (Invitrogen, 15596018CN, USA). RNA sequencing was performed by Genery Biotech (Shanghai, China) on the Illumina NovaSeq 6000 platform, generating 150-bp paired-end reads.

**Construction of *fam49a*-MO Zebrafish**

AB strain zebrafish (purchased from Nanjing Yaoshunyu Company) were raised in 28°C and 14h light/10h dark cycle. *Fam49a* expression was knocked down using morpholino antisense oligonucleotides (*fam49a*-MO) (**Table S3**). Embryos were collected and 8 ng/nL of *fam49a*-MO were injected to specifically block the translation of *fam49a* in zebrafish.

**Alcian blue staining**

Zebrafish embryos were collected at 120 hours post-fertilization (hpf) and fixed in ethanol overnight. After staining with Alcian blue (A8140, Solarbio, Beijing, China) for 24h, the embryos were bleached in a 1:1 mixture of 3% hydrogen peroxide and 2% potassium hydroxide solution until the bone structures were clearly visible. Finally, the specimens were transferred to 100% glycerol for preservation and imaging.

**In silico analysis**

Chromatin accessibility assay data of human embryonic stem cells (H1-hESCs) were obtained from Gene Expression Omnibus GSE18927. H3K4me1, H3K27ac and H3K4me3 ChIP-seq data of H1-hESCs were obtained from GSE16356. RNA-seq of 42 NSCL/P patients’ orbicularis oris muscle were obtained from GSE85748. HLTF ChIP-Seq on human K562 cells were obtained from GSE91466. The ChIP-seq data of CTCF for H1-hESC, HSMM, GM78, K562, HUVEC, IMR90 cells were obtained from GSE31477, GSE29611, and GSE33213. Topologically associating domain (TAD) and H1-hESC CTCF looping data were acquired from GSE69647. Hi-C data of H1-hESCs were downloaded from the ENCODE. Zebrafish scRNA-seq clustering data were obtained from the ZESTA website (https://db.cngb.org/stomics/zesta/)^[5]^.

GO and Reactome enrichment analysis were performed by the KOBAS online tool, and the top 10 pathways were selected for mapping (http://bioinfo.org/kobas/genelist/)^[6]^.

**Statistical analysis**

Comparisons between two groups were analyzed by Student’s *t*-test, while differences among multiple groups were assessed by one-way analysis of variance (ANOVA). Correlation analysis was employed to analyze the relationships between the transcription factors (TF) and *FAM49A* based on Pearson correlation coefficient. A **two-tailed** *P*<0.05 was considered statistically significant.

**
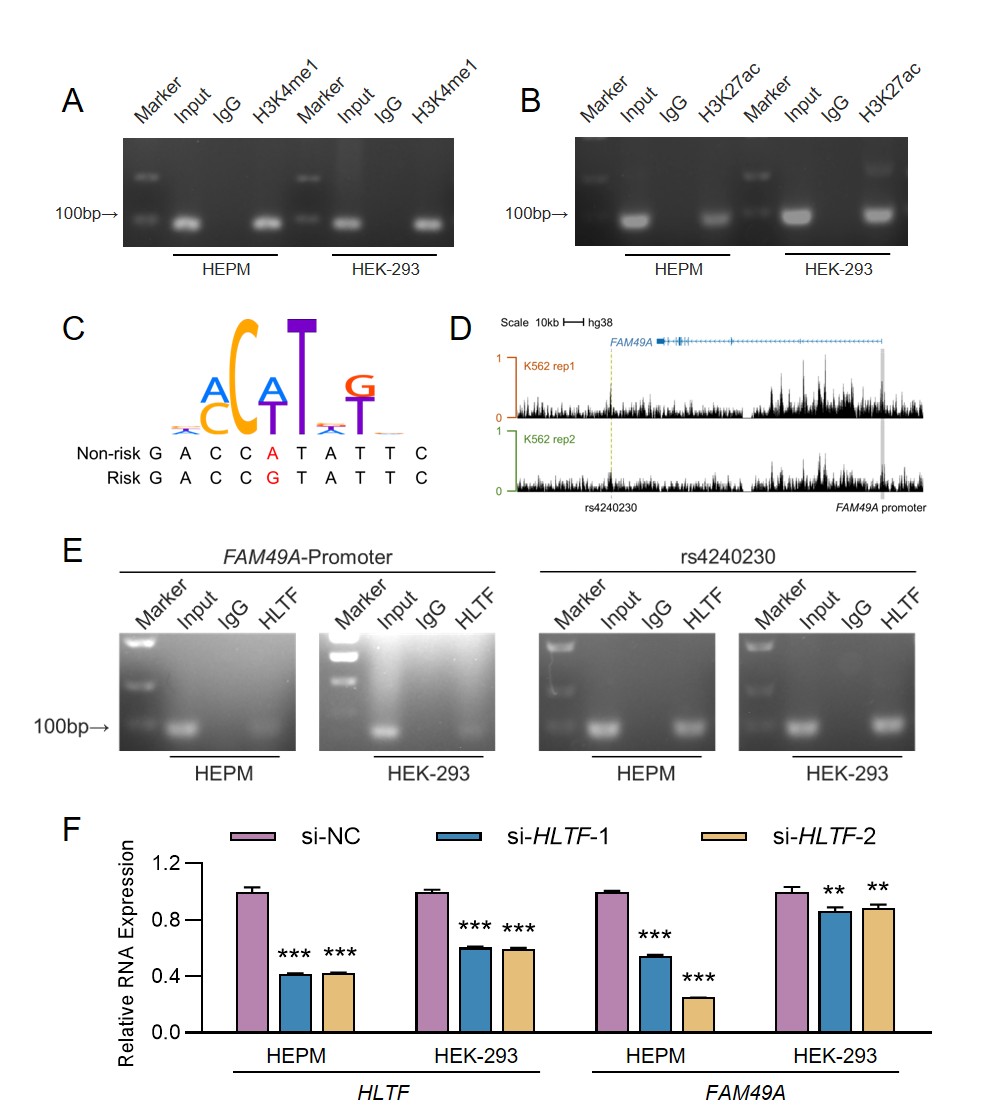
Additional results**

**Supplementary Figure S1. rs4240230 risk G allele recruited less HLTF, and decreased the expression of *FAM49A*.**

(A-B) ChIP gel electrophoreses illustrated the enrichment of H3K4me1 (A) and H3K27ac (B) on rs4240230 in HEPM and HEK-293 cells.

(C) Base sequence diagram of the rs4240230 region from PERFECTOS-APE platform.

(D) UCSC tracks of HLTF ChIP-seq data in K562 cells from GEO database (GSE91466) showed enrichment of HLTF at the rs4240230 locus and promoter region of *FAM49A*.

(E) ChIP gel electrophoreses illustrated the enrichment of HLTF on *FAM49A* promoter region and rs4240230 in HEPM and HEK-293 cells.

(F) *HLTF* was knocked down in HEPM and HEK-293 cells, and RT-qPCR was used to verify the expression of *FAM49A*.

Results were presented as mean±SD of three independent experiments. ***P* <0.01 or ****P* <0.001 indicates significant difference between the groups.


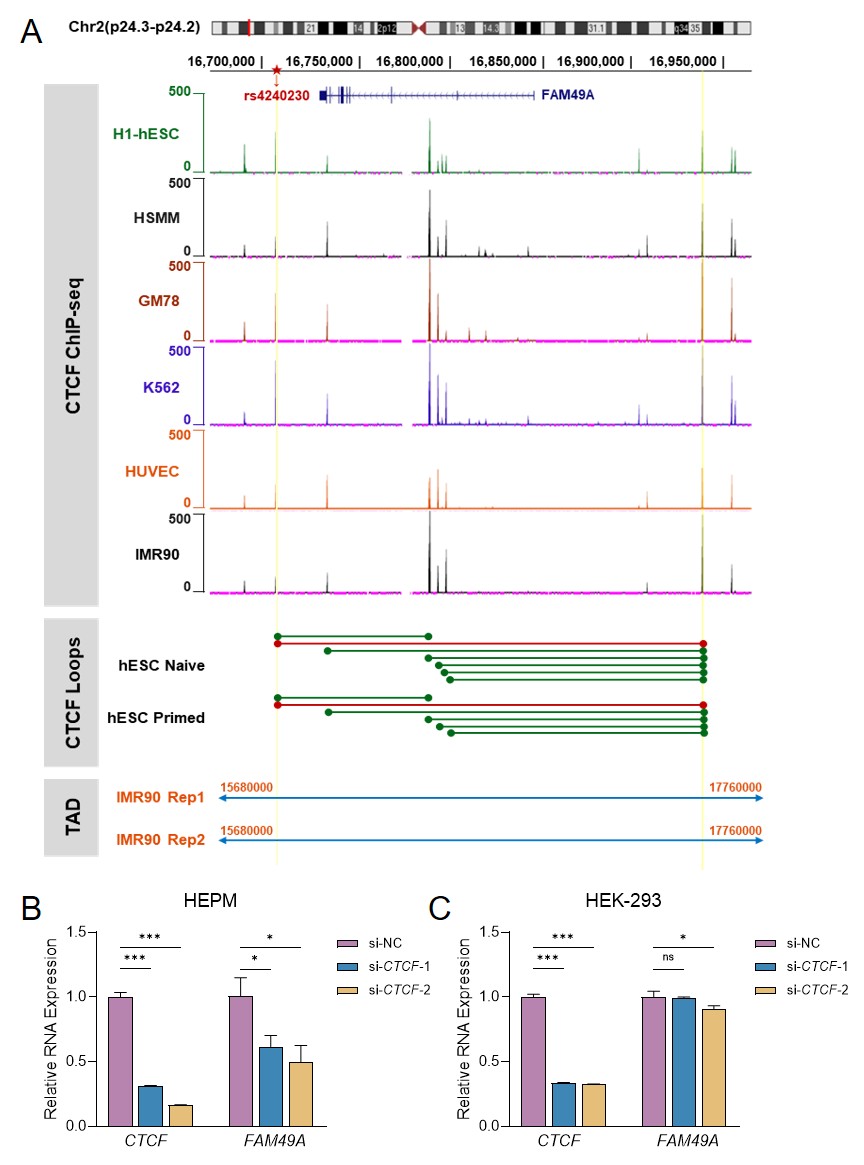


**Supplementary Figure S2. CTCF-mediated chromatin loop bridges rs4240230 and *FAM49A* promoter.**

(A) UCSC tracks of CTCF ChIP-seq data from six cell lines (GSE31477, GSE29611, GSE33213) demonstrate CTCF binding sites surrounding the rs4240230 locus and the *FAM49A* promoter. CTCF-mediated chromatin looping was identified by ChIA-PET in hESCs (GSE69647). Predicted TAD in hESCs are annotated (GSE69647).

(B-C) *CTCF* was knocked down in HEPM and HEK-293 cells (B), and *FAM49A* expression (C) was detected by RT-qPCR.

Results were presented as mean±SD of three independent experiments. ns, not significant, **P*<0.05 or ****P* <0.001 indicates a significant difference between the groups.


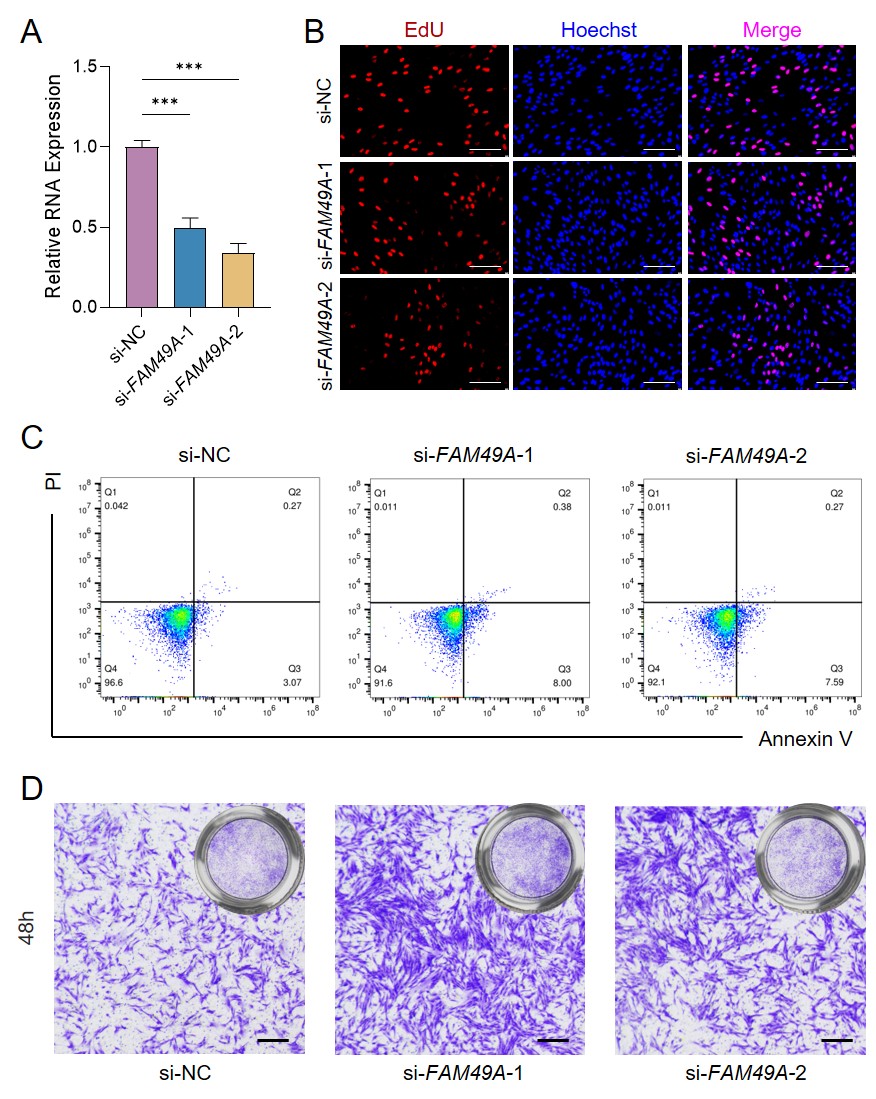


**Supplementary Figure S3.** ***FAM49A* knockdown affects HEPM cell proliferation, apoptosis and migration.**

(A) The efficiency of *FAM49A* knockdown in HEPM cells was detected by RT-qPCR.

(B) Representative fluorescence microscopy images showing EdU incorporation (proliferation) in HEPM cells.

(C) Representative flow cytometry dot plots analyzing apoptosis in HEPM cells.

(D) Representative images of migrated HEPM cells on the lower membrane surface in transwell assays.

Results were presented as mean±SD of three independent experiments. ns, not significant, ****P* <0.001 indicates a significant difference between the groups. Scale bar: 100 μm.


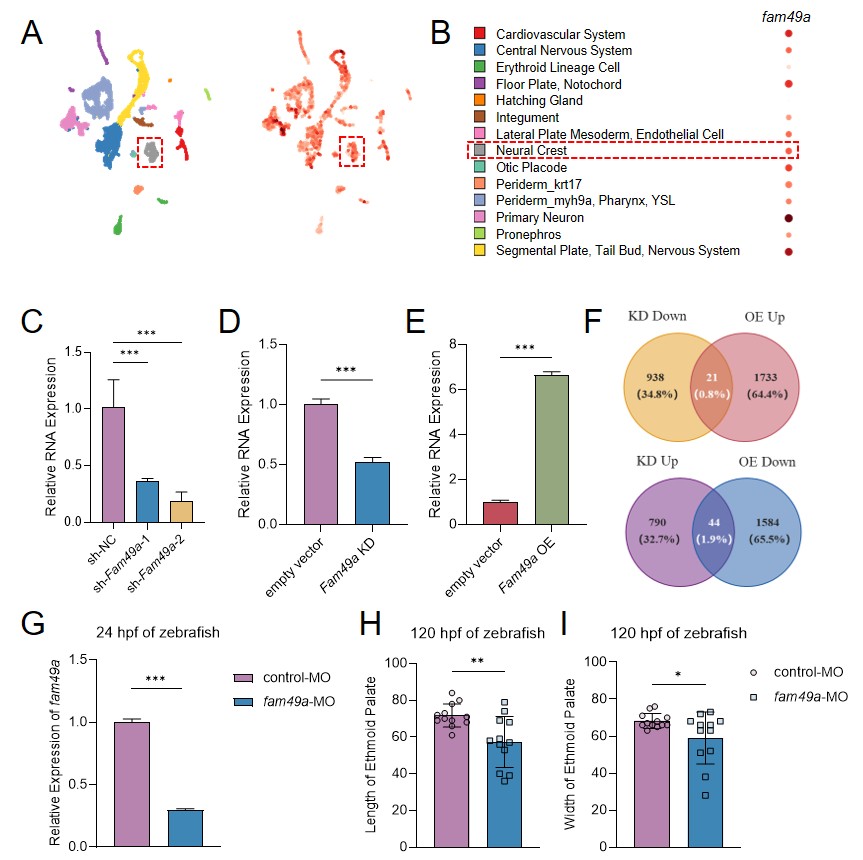


**Supplementary Figure S4. Investigating *Fam49a* function using O9-1 cells RNA-seq and zebrafish *in vivo* Assays.**

(A) uMAP visualization of scRNA in 18 hpf zebrafish and *fam49a* expression in different cell subclusters (https://db.cngb.org/stomics/zesta/).

(B) Heatmap of *FAM49A* expression across different cell types (https://db.cngb.org/stomics/zesta/).

(C) The efficiency of *Fam49a* knockdown in O9-1 cells was detected by RT-qPCR.

(D-E) shRNA-mediated knockdown (F) and lentiviral overexpression (G) efficiency of *Fam49a* in O9-1 cells detected in RNA-seq.

(F) DEGs exhibiting expression levels consistently correlated with *Fam49a* were identified through RNA-seq analysis.

(G) *fam49a* expression levels in 24 hpf embryos following microinjection of *fam49a*-MO or control-MO were detected by RT-qPCR.

(H-I) Statistical analysis of the length (J) and width (K) of ethmoid in 120 hpf zebrafish (each group, n=12).

Results were presented as mean±SD of three independent experiments. **P*<0.05, ***P*<0.01 or ****P* <0.001 indicates a significant difference between the groups.

**
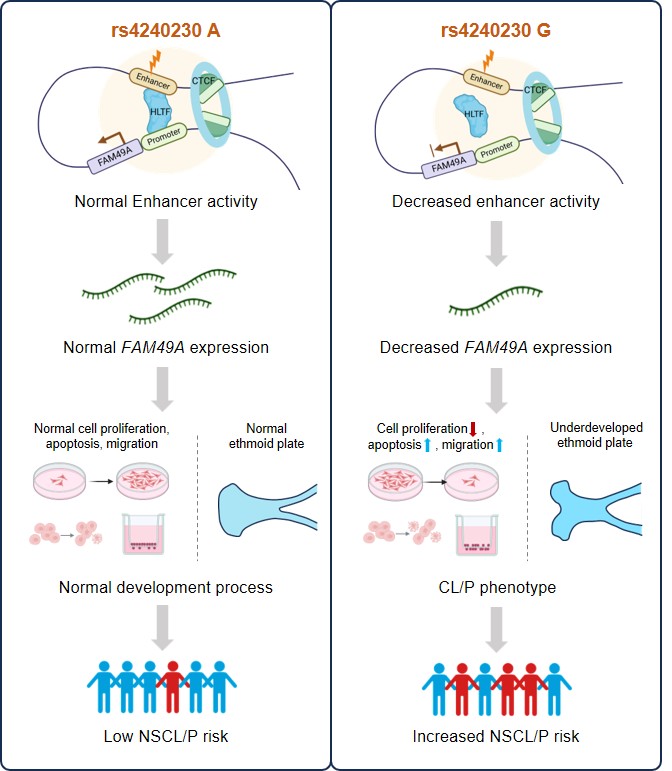
**

**Supplementary Figure S5. Graphical representation.**

Rs4240230 mutation contributes to NSCL/P pathogenesis, potentially through modulating HLTF binding affinity, which distally regulates *FAM49A* expression and subsequently disrupts craniofacial development.

| **Supplementary Table S1.** Bayesian Colocalization result of *FAM49A* | | | | | | |
| --- | --- | --- | --- | --- | --- | --- |
| Gene | Chr | PP0 | PP1 | PP2 | PP3 | PP4 |
| *FAM49A* | 2 | 1.46×10^−21^ | 4.62×10^−17^ | 6.08×10^−6^ | 0.191 | **0.809** |

**Supplementary Table S2.** Transcription factors binding on rs4240230 site predicted by PERFECTOS-APE

| Motif | Binding sequence | *P* A^a^ | *P* G^b^ | Fold change |
| --- | --- | --- | --- | --- |
| FOXC1 | GGACC[A/G]TA | 0.050642 | 0.003794 | 13.35 |
| TAL1+TCF3 | GGACC[A/G]TATTCT | 0.000438 | 0.005814 | 13.28 |
| YY1 | TGGACC[A/G]TATTC | 0.000507 | 0.005537 | 10.92 |
| USF1 | GACC[A/G]TATTCT | 0.003569 | 0.035358 | 9.91 |
| HLTF | GACC[A/G]TATTC | 0.001343 | 0.010952 | 8.15 |
| TEAD1 | CTGTGGACC[A/G]TA | 0.010954 | 0.001344 | 8.15 |
| MAX | CC[A/G]TATTCTA | 0.009444 | 0.0635 | 6.72 |
| FOSL2 | GTGGACC[A/G]TAT | 0.008177 | 0.054822 | 6.7 |
| TAL1+GATA1 | GGCATCCCTGTGGACC[A/G]T | 0.009945 | 0.066674 | 6.7 |

a: *P* for the match between transcription factor binding motifs and site motifs with rs4240230-A;

b: *P* for the match between transcription factor binding motifs and site motif

| **Supplementary Table S3.** Oligonucleotides and plasmid constructs used in this study | |
| --- | --- |
| **Primers for ChIP-PCR** | **Primers (5'-3')** |
| rs4240230 | F: GCATCCCTGTGGACCATATTCT  R: GTTAGCCCTGAGTGCCCATAAG |
| *FAM49A*-promoter | F: TGGGAGCGAGACTGGGTTCA  R: CACTTGGCCTTGCCTTCCCA |
| **Primers for qPCR** |  |
| **gene** | **Sequences (5'-3')** |
| *Gapdh* | F: AGGTCGGTGTGAACGGATTTG  R: TGTAGACCATGTAGTTGAGGTCA |
| *gapdh* | F: CCAACTGCCTGGCTCCTT  R: CCCATCAACGGTCTTCTGTG |
| *FAM49A* | F: TCAGTCGCAACCGCATCAAC  R: TGAGGCAGTCTGTGGTGTTCT |
| *Fam49a* | F: CTCACAGACTTACAGGCTTACA  R: CGGATGGAGAATTCGTAAAACC |
| *fam49a* | F: CCGAGTCGCTGGAGTTGA  R: GGTGCTGAGTGGGAGTGT |
| *CTCF* | F: ATGTGCGATTACGCCAGTGTA  R: TGAAACGGACGCTCTCCAGTA |
| *HLTF* | F: TTTTCCACGCCTCTCATATCCA  R: AGCGTAGTCCAACCACATGAC |
| **Primers for** **3C** |  |
| **Restriction site** | **Sequences (5'-3')** |
| N1 | F: AGGTGGACAGATTAAGCCAGAA |
| N2 | F: TCCGAGGCCGTCTCGCAG |
| N3 | F: ACACTCCCCTTCCCACCACC |
| N4 | F: ACCTTGCCGTCACCCTCTCT |
| N5 | F: TAGCCGTGCTGCTTTGGGAA |
| N6 | F: AGAGCCGCACCTGTCTCTGA |
| N7 | F: TCGAGCAATGCTTCAGTAGAGC |
| N8 | F: AGCCATAGGTGTGAGTGTTGGC |
| N9 | F: AACCATGGCTGAACACGGCA  R: TGACCCTCAATTTCCCCATCCT |
| N10 | R: ACCCCTTTCCCCCTTAAGCA |
| N11 | R: GCAGTTCCACCATACACACGGT |
| **Target genes** | **siRNA (5'-3')** |
| *FAM49A* | si-*FAM49A*-1:  F: CAAUGACAAGUGUCUGUAAAGTT  R: CUUUACAGACACUUGUCAUUGTT |
| *FAM49A* | si-*FAM49A*-2:  F: GGUUCACUACAAAGCACUUGATT  R: UCAAGUGCUUUGUAGUGAACCTT |
| *CTCF* | si-*CTCF*-1:  F: GGUGGAGACACUAGAACAATT  R: UUGUUCUAGUGUCUCCACCTT |
| *CTCF* | si-*CTCF*-2:  F: GUGCAAUUGAGAACAUUAUTT  R: AUAAUGUUCUCAAUUGCACTT |
| *HLTF* | si-*HLTF*-1:  F: GCAUUACAACGAGAUCCUATT  R: UAGGAUCUCGUUGUAAUGCTT |
| *HLTF* | si-*HLTF*-2:  F: GCAAAGAACUUCCACCAUUTT  R: AAUGGUGGAAGUUCUUUGCTT |
| Negative Control | si-NC:  F: UUCUCCGAACGUGUCACGUTT  R: ACGUGACACGUUCGGAGAATT |
| **Target genes** | **shRNA (5'-3')** |
| *Fam49a* | sh-*Fam49a*-1:  F: CAAUGACAAGUGUCUGUAAAGTT  R: CUUUACAGACACUUGUCAUUGTT |
| *Fam49a* | sh-*Fam49a*-2:  F: GGUUCACUACAAAGCACUUGATT  R: UCAAGUGCUUUGUAGUGAACCTT |
| Negative Control | sh-NC:  F: UUCUCCGAACGUGUCACGUTT  R: ACGUGACACGUUCGGAGAATT |
| **Targets** | **sgRNA (5'-3')** |
| rs4240230-containing genomic region | sgRNA-1: ATTAATAGAATATGGTCCAC  sgRNA-2: TTAATAGAATATGGTCCACA |
| Control | sgNC: GTGTAGTTCGACCATTCGTG |
| **Probes for** **EMSA** |  |
| **Labeled probes** | **Sequences (5'-3')** |
| rs4240230-A | F: TCCCTGTGGACCATATTCTATTAAT  R: ATTAATAGAATATGGTCCACAGGGA |
| rs4240230-G | F: TCCCTGTGGACCGTATTCTATTAAT  R: ATTAATAGAATACGGTCCACAGGGA |
| **Probes for** **EMSA** |  |
| **Competing probes** | **Sequences (5'-3')** |
| rs4240230-A | F: TCCCTGTGGACCATATTCTATTAAT  R: ATTAATAGAATATGGTCCACAGGGA |
| rs4240230-G | F: TCCCTGTGGACCGTATTCTATTAAT  R: ATTAATAGAATACGGTCCACAGGGA |
| **Plasmids for luciferase reporter assay** | **Sequences (5'-3')** |
| *FAM49A*-promoter | GCAGCTTCGGGCACGTACCTTGGCACACTATGGGGATTTAACACATGTTTCATGATGTTCTGGCTGATGGACTGGGAAAGGAAGCACCCGAAGCCGGGGTCCCCAGGAGCTCTCCCTCCCCTCAACAGGAGCTCTGACTCACAACCTGTTTGGCACTGCTGAGTCCTCTCCGGTTTCCTGAATCTCCCTCTCTCTAATGCAAAAAGCCTCCATGGCAGATGTTTTAGAACAATGATCACGTCAATGACGCTGTAACCCACTACTTTCTTTTTGCATCATAGAATTTTTTAAGTGTTTTTGCAGACTTACTCTTTAAGGTTAATTACATGTTTTGGATTGAGCCTTTGGGAAGACTTTACAGTGGTCCTATCGGATAACTGCGGGCCCCAAGGCTGGAGATCTGTTAATAGGGTGGGCAGAAAGGGACTGGGAGCGAGACTGGGTTCAGAATCTCAAGTAATTTGGAGTGTGCCTAGGGATTGGGATGATTTGGCTGGGCATTAATTTGAAGGTGAATATGCGTCTAGAGATTGCGGGGATTTCTTTTTCTTGGAGGGCAACCGTGGGTGTGGAGGAATTTGGTGTGCAGAAACAGAAAGGTCTGGTTCGGGCTTCCTCTGGGAAGGCAAGGCCAAGTGGCCAAGGCTGAGAAGGGGCAGGGGCGACCGTGCTGGGGCGGGTCCGCACCAGCTCCTGGGAGCAGAGAGCAGAGCCGGGCAAAGAGATGGGGTTGTCCAGGCTAGGGTGAGGACGCGGCCGGTGACAGCGCGGGTGGGGATCCCCCGGGGCCGGGGCGGGGCGGCTGCCGCGAGCCGGGCTGGGGGCGGGGCGGGGCGGGGAGGGACGGGGAGGGGCGGGGCGGGGCTCCGAGGCCGTCTCGCAGGCTGCGGCGGCCGCGGGAGAGGCGTACTCGGCGGCGGCGGAGCGGGCGGCAGAGCAGGGCGGCGGCGACTCGGTGAGCGCGGCGCGGCTGGGGGCGCGGCAGGGGCGCGGGCGGGGG |
| rs4240230(A/G)-Enhancer | aatgaaaggcagcgagttcagtaggaaaaattcatgttacatccaaaaatctcatttgaactctaactgtgtttaccaggtgtgggactctgaattagtaaacacctttgaccctcaatttccccatcctttaaatgaagttaataattggaggaattaaataaggtaataaaaaatgtacaggcgatatgacaaaacgtagaacgttcgttactatcataataattaatgctgttgtgcaaatagcaatttactgttttatagtttcgcatccacctttcattgcttgctctgtgataatggagagggaccctgtaaacgtgtccgccttgccactttgtgcaatatttagctccttcagtagagggcgctgcaggaacatgtcaggagggggccttctcttccggattctggattctggtatacacttggagcgtggcatttgggggacagccactggtgcgcagccccatcaagttttggtggcatccctgtggacc(A/G)tattctattaatcttcagtggtacccctgtggcagatccccagtgattcttatgggcactcagggctaactccccagccttgcctgcactgtgacgggtttccagcttaccagtcatagtgtgtgtaactaactgcctgccagctttggcccacatgcaccccagaggtgcatttcttgctttccagttcgttctgtgagtctctggccacctgtgtcagcctacctgtatctgaggcgggtattttttttttctgccgtgttcagccatggttccctgatcatcagcctcagtccagtatctgtggactagctccaacgtggggtgacttagggaaattcctggccatccagcagagtgcaaaccccaccttcccaatgaagggagtccaaccttggggaagagacccttccttccatgtttgcttcttttttaaatgttctccatcaatcttaaggaatcctttcaagtattttttactcctttacaggttaactgca |
| **MO for zebrafish** |  |
| **Targets** | **Sequences (5'-3')** |
| *fam49a* | ATATGAGCAGCGCACCTAGTTTCAA |
| control | CCTCTTACCTCAGTTACAATTTATA |

**References**

1. Lou S, Zhu G, Xing C, Hao S, Lin J, Xu J, Li D, Du Y, Mi C, Sun L, Wang L, Wang M, Du M, Pan Y. Transcriptome-wide association identifies KLC1 as a regulator of mitophagy in non-syndromic cleft lip with or without palate. 2024, **3**(6)**:** e262.

2. Vosa U, Claringbould A, Westra HJ, Bonder MJ, Deelen P, Zeng B, Kirsten H, Saha A, Kreuzhuber R, Yazar S, Brugge H, Oelen R, de Vries DH, van der Wijst MGP, Kasela S, Pervjakova N, Alves I, Fave MJ, Agbessi M, Christiansen MW, Jansen R, Seppala I, Tong L, Teumer A, Schramm K, Hemani G, Verlouw J, Yaghootkar H, Sonmez Flitman R, Brown A, Kukushkina V, Kalnapenkis A, Rueger S, Porcu E, Kronberg J, Kettunen J, Lee B, Zhang F, Qi T, Hernandez JA, Arindrarto W, Beutner F, Consortium B, i QTLC, Dmitrieva J, Elansary M, Fairfax BP, Georges M, Heijmans BT, Hewitt AW, Kahonen M, Kim Y, Knight JC, Kovacs P, Krohn K, Li S, Loeffler M, Marigorta UM, Mei H, Momozawa Y, Muller-Nurasyid M, Nauck M, Nivard MG, Penninx B, Pritchard JK, Raitakari OT, Rotzschke O, Slagboom EP, Stehouwer CDA, Stumvoll M, Sullivan P, t Hoen PAC, Thiery J, Tonjes A, van Dongen J, van Iterson M, Veldink JH, Volker U, Warmerdam R, Wijmenga C, Swertz M, Andiappan A, Montgomery GW, Ripatti S, Perola M, Kutalik Z, Dermitzakis E, Bergmann S, Frayling T, van Meurs J, Prokisch H, Ahsan H, Pierce BL, Lehtimaki T, Boomsma DI, Psaty BM, Gharib SA, Awadalla P, Milani L, Ouwehand WH, Downes K, Stegle O, Battle A, Visscher PM, Yang J, Scholz M, Powell J, Gibson G, Esko T, Franke L. Large-scale cis- and trans-eQTL analyses identify thousands of genetic loci and polygenic scores that regulate blood gene expression. Nat Genet 2021, **53**(9)**:** 1300-1310.

3. Liu B, Gloudemans MJ, Rao AS, Ingelsson E, Montgomery SB. Abundant associations with gene expression complicate GWAS follow-up. Nat Genet 2019, **51**(5)**:** 768-769.

4. Li X, Tian Y, Qiu L, Lou S, Zhu G, Gao Y, Ma L, Pan Y. Expression Quantitative Trait Locus Study of Non-Syndromic Cleft Lip with or without Cleft Palate GWAS Variants in Lip Tissues. Cells 2022, **11**(20).

5. Liu C, Li R, Li Y, Lin X, Zhao K, Liu Q, Wang S, Yang X, Shi X, Ma Y, Pei C, Wang H, Bao W, Hui J, Yang T, Xu Z, Lai T, Berberoglu MA, Sahu SK, Esteban MA, Ma K, Fan G, Li Y, Liu S, Chen A, Xu X, Dong Z, Liu L. Spatiotemporal mapping of gene expression landscapes and developmental trajectories during zebrafish embryogenesis. Dev Cell 2022, **57**(10)**:** 1284-1298 e1285.

6. Bu D, Luo H, Huo P, Wang Z, Zhang S, He Z, Wu Y, Zhao L, Liu J, Guo J, Fang S, Cao W, Yi L, Zhao Y, Kong L. KOBAS-i: intelligent prioritization and exploratory visualization of biological functions for gene enrichment analysis. Nucleic Acids Res 2021, **49**(W1)**:** W317-W325.
